# Aktuelle Möglichkeiten und Herausforderungen bei der Diagnostik des laryngopharyngealen Refluxes 

**DOI:** 10.1007/s00106-021-01006-3

**Published:** 2021-02-22

**Authors:** D. Runggaldier, J. Hente, M. Brockmann-Bauser, D. Pohl, J. E. Bohlender

**Affiliations:** 1 Klinik für Otorhinolaryngologie, Head and Neck Surgery, Abt. für Phoniatrie und klinische Logopädie, Universitätsspital Zürich, Frauenklinikstrasse 24, 8091 Zürich, Schweiz; 2 Gastroenterologie und Hepatologie, UniversitätsSpital Zürich, Rämistrasse 100, 8091 Zürich, Schweiz; 3 Universität Zürich, Rämistrasse 71, 8006 Zürich, Schweiz; 4 Klinik für ORL, Universitätsspital Zürich, Frauenklinikstrasse 24, 8091 Zürich, Schweiz

**Keywords:** Reflux Symptom Index (RSI), Reflux Finding Score (RFS), Ösophageale 24h Impedanz pH Metrie (24h-MII-pH-Metrie), Oropharyngeale 24h pH Metrie (Restech), Pepsin, Reflux Symptom Index (RSI), Reflux Finding Score (RFS), Esophageal 24-hour pH impedance Reflux Monitoring, Oropharyngeal 24h pH Monitoring (Restech), Pepsin

## Abstract

Der laryngopharyngeale Reflux (LPR) ist definiert als ein Zurückfließen von gastralem bzw. gastroduodenalem Sekret in den Larynx- bzw. Pharynxbereich und ist durch ein sehr breites Spektrum an teils unspezifischen Symptomen wie beispielsweise chronischem Husten, zervikalem Globusgefühl oder Hypersekretion von Mukus im Larynx und Pharynx charakterisiert. Aufgrund des Fehlens eines Goldstandards und der heterogenen Studienlage gestaltet sich die Diagnosestellung des LPR jedoch weiterhin schwierig und wird in absehbarer Zeit weiterhin kontrovers diskutiert werden. Insgesamt kann man jedoch davon ausgehen, dass bei suggestiver Anamnese mit erhöhten Scores im Reflux Symptom Index (RSI), entsprechenden endoskopischen Befunden mit pathologischem Reflux Finding Score (RFS) und auffälliger ösophagealer oder oropharyngealer 24-h-pH-Metrie ohne Hinweise auf eine andere Grunderkrankung die Diagnose eines LRP wahrscheinlich ist. In der vorliegenden Übersichtsarbeit sollen die genannten Methoden ebenso wie neuere Werkzeuge bei der Diagnose des LPR kritisch diskutiert werden.

## Abstract (en)

Laryngopharyngeal reflux (LPR) is defined as backflow of gastral or gastroduodenal content into the upper aerodigestive tract and characterized by a variety of unspecific symptoms such as chronic cough, globus sensation, or mucus hypersecretion. Due to the lack of a gold standard and the heterogeneity of studies, the diagnosis of LPR is still problematic and challenging. However, in patients with characteristic symptoms and endoscopic findings, with an increased reflux symptom index, a pathologic reflux finding score (RFS), pathologic 24 h esophageal or oropharyngeal pH monitoring, and without any other underlying condition, the diagnosis of LPR is probable. In the following review, we critically discuss the abovementioned methods as well as more recent tools such as measurements of pepsin concentrations in the saliva for diagnosis of LPR.

Als Krankheitsbild wird der laryngopharyngeale Reflux (LPR) wissenschaftlich erst seit 1991 systematisch untersucht [1] und ist definiert als ein Zurückfließen von gastralem bzw. gastroduodenalem Sekret in den oberen aerodigestiven Trakt, insbesondere in den Larynx- und Pharynxbereich [2]. Eine genauere Abschätzung der Prävalenz sowie Inzidenz dieser Erkrankung ist dabei auch im Hinblick auf das Fehlen eines diagnostischen Goldstandards sowie die heterogene epidemiologische Studienlandschaft bisher sehr problematisch [1–4]. Zudem stand in der Vergangenheit bei der Abklärung des LPR häufig nur eine rein ösophageale pH-Metrie mit Abklärung der rein sauren Refluxkomponente zur Verfügung, sodass epidemiologische Rückschlüsse bei oftmals fehlender Berücksichtigung von nichtsauren oder gemischten Refluxereignissen weiter erschwert sind [5].

Pathomechanistisch kann die Exposition gegenüber saurem, aber auch nichtsaurem Reflux einerseits eine direkte Schädigung und Entzündung der Schleimhäute im oberen aerodigestiven Trakt zur Folge haben und dabei zu den charakteristischen LPR-assoziierten Larynx- und Pharynxveränderungen führen [2]. Auf der anderen Seite werden in der Literatur in den letzten Jahren weitere Pathomechanismen beschrieben, die auf einer Reizung von neuronalen Reflexbögen v. a. im distalen Ösophagus beruhen und als Trigger von chronischem Husten, zervikalem Globusgefühl oder Hypersekretion von Mukus im Larynx und Pharynx fungieren können [6–8]. Insgesamt kann sich der LPR daher durch ein sehr breites Spektrum an teils sehr unspezifischen Symptomen präsentieren. Dieses kann neben den genannten Beschwerden unter anderem noch Heiserkeit bis hin zu einem hyperreagiblen Larynx, „postnasal drip“, aber auch klassische Symptome des gastroösophagealen Refluxes (GERD) wie retrosternales Brennen oder saures Aufstoßen beinhalten [9].

Besteht nun bei entsprechender Anamnese und suggestiver Symptomatik mit gleichzeitig fehlenden Hinweisen auf eine andere Grunderkrankung der Verdacht auf einen LPR, so stehen trotz Fehlen eines etablierten Goldstandards bei der Diagnose des LPR zahlreiche Werkzeuge zu Verfügung, die in der nachfolgenden Übersichtsarbeit zusammengefasst und kritisch diskutiert werden.

## Diagnostische Möglichkeiten

### Fragebögen

Der Reflux Symptom Index (RSI) ist der älteste und auch bekannteste LPR-Symptomfragebogen mit insgesamt 9 Items (Tab. 1). Dieser wurde bisher zwar in zahlreichen Studien eingesetzt und validiert [9–12], dennoch haben sich in den letzten Jahren diesbezüglich einige Schwachpunkte herauskristallisiert: So sind die im RSI aufgelisteten Beschwerden einerseits unspezifisch und werden oftmals auch von Patienten mit anderen Erkrankungen wie beispielsweise einer chronischen Rhinosinusitis, Allergien oder chronischen oberen Atemwegsinfekten ohne pH-metrisch bestätigten Reflux beklagt. Auf der anderen Seite finden weitere mit LPR assoziierte Symptome wie etwa die Odynophagie oder Schmerzen im Hals- und Pharynxbereich im RSI keine Beachtung [2]. Zudem werden im RSI lediglich die Symptomstärke, nicht jedoch die Häufigkeit der entsprechenden Symptome und die damit einhergehende Beeinträchtigung der Lebensqualität erfasst [2]. Im Hinblick darauf wurde daher 2018 im „international project of young otolaryngologists of the International Federation of Otorhinolaryngological Societies“ ein neuer Reflux Symptom Score (RSS) aufgestellt, der eine genauere Differenzierung der Symptomatik erlaubt und der in den kommenden Jahren in zahlreichen Kliniken weiter untersucht und validiert werden soll (Tab. 2; [12, 13]).

**Table 1:** 

The Reflux Symptom Index						
Within the last month, how did the following problems affect you?	0	1	2	3	4	5
Hoarseness or a problem with your voice						
Clearing your throat						
Excess throat mucus or postnasal drip						
Difficulty swallowing food, liquids or pills						
Coughing after you ate or after lying down						
Breathing difficulties or chocking episodes						
Troublesome or annoying cough						
Sensation of something sticking in your throat or a lump in your throat						
Heartburn, chest pain, indigestion or stomach acid coming up

**Table 2:** 

Reflux Symptom Score			
Within the last month, I suffered from one or several followed symptoms:	Disorder Frequency	Disorder Severity	Quality of life impact
Hoarseness or voice problem	0‑1-2-3-4‑5	0‑1-2-3-4‑5	0‑1-2-3-4‑5
Throat pain or pain during swallowing time	0‑1-2-3-4‑5	0‑1-2-3-4‑5	0‑1-2-3-4‑5
Difficulty swallowing (pills, liquids or solid foods)	0‑1-2-3-4‑5	0‑1-2-3-4‑5	0‑1-2-3-4‑5
Clearing your throat	0‑1-2-3-4‑5	0‑1-2-3-4‑5	0‑1-2-3-4‑5
Sensation of something sticking in the throat	0‑1-2-3-4‑5	0‑1-2-3-4‑5	0‑1-2-3-4‑5
Excess mucus in the throat or post nasal drip sensation	0‑1-2-3-4‑5	0‑1-2-3-4‑5	0‑1-2-3-4‑5
Halitosis	0‑1-2-3-4‑5	0‑1-2-3-4‑5	0‑1-2-3-4‑5
Heartburn, stomach acid coming up, regurgitations	0‑1-2-3-4‑5	0‑1-2-3-4‑5	0‑1-2-3-4‑5
Abdominal pain or diarrheas	0‑1-2-3-4‑5	0‑1-2-3-4‑5	0‑1-2-3-4‑5
Indigestion, abdominal distension or flatus	0‑1-2-3-4‑5	0‑1-2-3-4‑5	0‑1-2-3-4‑5
Cough after eating or lying down or daytime cough	0‑1-2-3-4‑5	0‑1-2-3-4‑5	0‑1-2-3-4‑5
Breathing difficulties, breathlessness or wheezing	0‑1-2-3-4‑5	0‑1-2-3-4‑5	0‑1-2-3-4‑5
–	–	*RSS total score:*	*Quality of life score:*

Ein häufig eingesetzter und bekannter Fragebogen auf Untersucherseite ist der sog. Reflux Finding Score (RFS), bei dem insgesamt 8 endoskopische Befunde des Larynx evaluiert und teils auch gemäß Schweregrad eingestuft werden (Tab. 3; [14]). Ähnlich wie im Fall des RSI wird auch dieses Instrument in den letzten Jahren zunehmend kontrovers diskutiert: Einerseits finden im RFS einige LPR-assoziierte Befunde wie beispielsweise Stimmlippenerytheme, Leukoplakien oder entzündliche Veränderungen der posterioren Pharynxwand keine Beachtung. Andererseits wird der Auswertung des RFS eine hohe Abhängigkeit vom jeweiligen Untersucher zugeschrieben, sodass auch hier kürzlich durch die Arbeitsgruppe „young otolaryngologists of the International Federation of Otorhinolaryngological“ das Reflux Sign Assignment (RSA) als neues Instrument entwickelt wurde [12]. Dieses erlaubt eine sehr detaillierte Differenzierung von LPR-assoziierten laryngealen und extralaryngealen Veränderungen. Ein Score > 14 kann als pathologisch betrachtet werden und ist mit dem Vorliegen eines LPR vereinbar. Auch konnte in ersten Validierungen bereits eine hohe Intra- unter Inter-Beurteiler-Reliabilität demonstriert werden [13].

**Table 3:** 

Reflux Finding Score	
Subglottic edema	0 = absent
	2 = present
Ventricular obstruction	0 = absent
	2 = present
	4 = complete
Erythema/hyperemia	0 = absent
	2 = arytenoid only
	4 = diffuse
Vocal fold edema	0 = absent
	1 = mild
	2 = moderate
	3 = severe
	4 = polypoid
Diffuse laryngeal edema	0 = absent
	1 = mild
	2 = moderate
	3 = severe
	4 = obstructing
Posterior commissure hypertrophy	1 = mild
	2 = moderate
	3 = severe
Granuloma/granulation tissue	0 = absent
	2 = present
Thick endolaryngeal mucus	0 = absent
	2 = present

### Apparative Diagnostik

**Figure 1:**
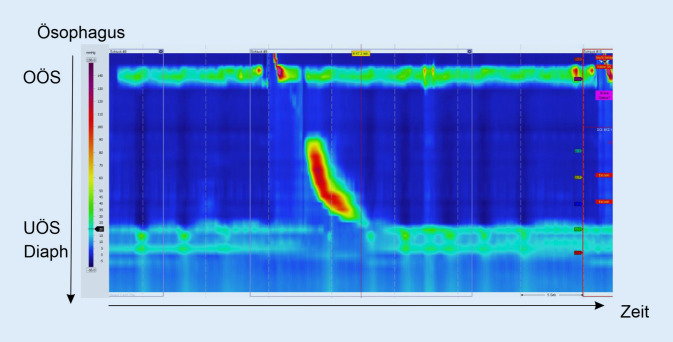

Besteht bei suggestiver Anamnese und endoskopischen Befunden der Verdacht auf einen LPR, kann dieser insbesondere bei Unklarheiten oder beispielsweise bei fehlendem Ansprechen auf eine säurehemmende Therapie durch eine weiterführende apparative Diagnostik weiter abgeklärt werden:

Dabei besteht einerseits auf gastroenterologischer Seite die Möglichkeit einer hochauflösenden Ösophagusmanometrie (HRM), gefolgt von einer kombinierten 24-h-Impedanz-pH-Metrie (24-h-MII-pH-Metrie, MII: Multikanal-intraluminale-Impedanzmessung). Die Ösophagusmanometrie ist erforderlich, um ösophageale Motilitätsstörungen oder bestimmte Risikofaktoren für eine Refluxerkrankung wie beispielsweise einen hypotonen unteren Ösophagussphinkter oder eine Hiatushernie abzuklären [15]. Ebenfalls kann in der gleichen Untersuchung der Ösophagus entlang seiner Längenausdehnung vermessen werden, damit folgend im Rahmen der 24-h-MII-pH-Metrie eine exakte Positionierung der pH-Elektrode, üblicherweise ca. 5 cm proximal des unteren Ösophagussphinkters, möglich ist (Abb. 1; [8]).

**Figure 2:**
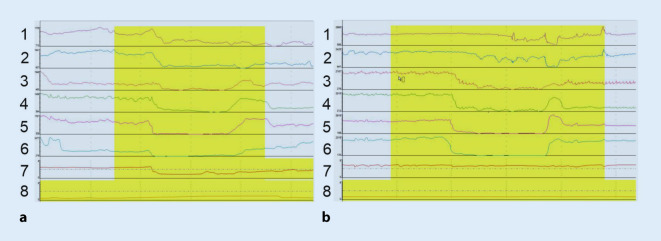

Bei der auf die Manometrie folgenden modernen 24-h-MII-pH-Metrie werden im Vergleich zu der früher durchgeführten reinen pH-Metrie im distalen Ösophagus mehrere diagnostische Vorteile erzielt. Auf der einen Seite verfügt ein MII-pH-Metrie-Katheter über mehrere „Impedanzelektroden“, die in unterschiedlich großen Abständen zur Katheterspitze platziert sind und entsprechende Veränderungen des Wechselstromwiderstands erfassen können. Dies ermöglicht schließlich eine exakte Detektion des intraösophagealen Transits von Flüssigkeiten und Gasen, sodass bei gleichzeitigem Vorhandensein einer pH-Elektrode im distalen Ösophagus saure, gemischte und nichtsaure Refluxereignisse gut voneinander abgegrenzt werden können (Abb. 2).

Allerdings gibt es in Bezug zum LPR bei der 24-h-MII-pH-Metrie, die primär im Rahmen der GERD-Diagnostik entwickelt wurde, keine allgemein anerkannten Interpretationskriterien. So bedienen sich beispielsweise einige Studien zur Diagnostik des LPR des DeMeester-Scores [16], der eigentlich in Hinblick auf den GERD etabliert wurde [17]. Hingegen wird bei anderen Studien als maßgebendes Kriterium für einen LPR der Abfall der Impedanz im Bereich der proximalsten Impedanzelektroden herangezogen [18]. Ein Nachteil der insgesamt eher aufwendigen 24-h-MII-pH-Metrie ist durch den schlechten prädiktiven Aussagewert bei Fehlen von klassischen GERD-Symptomen für das Ansprechen von LPR-typischen Symptomen, wie beispielsweise dem chronischen Husten auf eine säurehemmende Therapie, gegeben [19].

**Figure 3:**
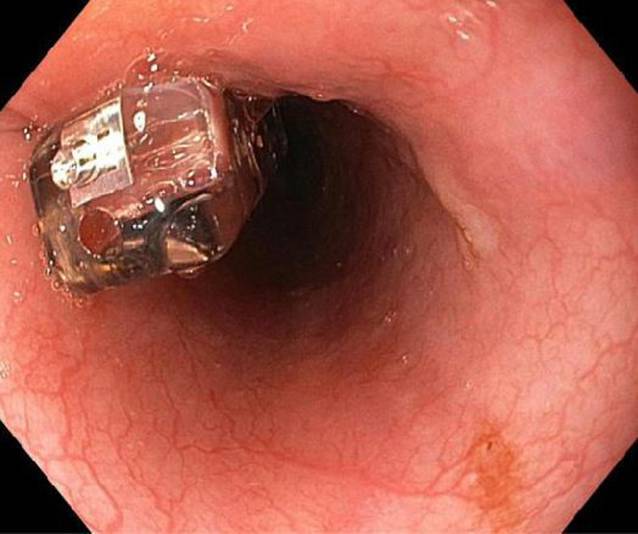

Eine weitere Möglichkeit, bestehende Refluxereignisse zu erfassen, stellt die Implantation einer drahtlosen 48-h-Bravo-Kapsel dar (Abb. 3). Diese eignet sich besonders für Patienten, welche eine 24-h-pH-Metrie-Sonde über die Nase nicht tolerieren können oder bei denen eine Säuremessung über einen längeren Zeitraum geplant ist. Hierbei wird im Rahmen einer Gastroskopie eine Einmalkapsel im distalen Ösophagus platziert, wobei unter endoskopischer Sicht eine Positionierung der Kapsel 6 cm proximal des unteren Ösophagussphinkters möglich ist. Diese misst über 48 h den vorliegenden pH-Wert, sendet die Ergebnisse per Funk an ein kleines Empfängergerät. Sie fällt nach wenigen Tagen von selbst ab und wird auf natürlichem Weg ausgeschieden. Im Vergleich zur konventionellen Messung fällt somit die möglicherweise störende transnasal eingelegte Sonde weg [20, 21]. Als nachteilig könnten bei dieser Methode jedoch höhere Kosten [22] sowie die fehlende Impedanzmessung zur Diagnostik von nichtsauren Refluxereignissen erachtet werden [23]. Hinsichtlich des diagnostischen Outcomes einer Refluxerkrankung unterscheiden sich die beiden Verfahren/Techniken nicht [24].

Aufseiten der Hals‑, Nasen‑, Ohrenheilkunde besteht die Möglichkeit der Durchführung einer speziellen oropharyngealen 24-h-pH-Metrie, welche von der Fa. Restech (11011 Brooklet Dr, Ste 300, Houston, TX, USA) vertrieben wird. Bei dieser Methodik wird ein dünner Katheter mit einer speziellen pH-Messelektrode transnasal bis in den Oropharynx vorgeschoben, wo saurer Reflux in flüssiger, aber auch in aerosolierter Form detektiert werden kann. Aus den Messergebnissen kann ein „Ryan Score“ berechnet werden, in dem sowohl die Anzahl der sauren Refluxepisoden als auch die prozentuale Zeit mit einem pH-Abfall unter die vordefinierten Grenzwerte und die Dauer der längsten sauren Refluxepisode im Pharynx Berücksichtigung finden. Ein Ryan Score in aufrechter Position von > 9,41 bzw. in liegender Position von > 6,8 kann für einen schweren sauren LPR sprechen [25]. Bislang erfolgte der Einsatz einer oropharyngealen pH-Metrie lediglich in wenigen Studien: Dabei wird der oropharyngealen pH-Metrie neben einer einfachen Durchführbarkeit und meist guter Tolerierbarkeit durch die Patienten eine höhere Sensitivität bei der Detektion von sauren Refluxepisoden zugeschrieben [26, 27]. Jedoch konnten weitere Studien keine Korrelation zwischen den Befunden aus der oropharyngealen pH-Metrie und dem RFS, RSI, der klassischen ösophagealen 24-h-MII-pH-Metrie oder den Pepsinspiegeln im Speichel identifizieren [18, 28–30]. Ebenfalls finden bei dieser Methodik gemischte und rein nichtsaure Refluxepisoden, die ebenfalls in die Pathogenese des LPR involviert sein können, keine Beachtung [29]. Insgesamt bleibt die Wertigkeit der oropharyngealen 24-h-pH-Metrie wie auch der anderen pH-metrischen Verfahren bei der Diagnosestellung des LPR unzureichend verstanden und gleichzeitig auch umstritten. Weitere Studien sind somit zur Klärung dieser Fragen, insbesondere auch zur Validierung von Normwerten und zur Etablierung von validen Diagnosekriterien in Bezug auf den LPR, erforderlich.

### Laborchemische Bestimmung von Pepsin im Speichel

Pepsin, das von den gastralen Hauptzellen als wichtiges proteolytisches Enzym produziert und in den Magensaft sekretiert wird, wird in der Literatur als sensitiver und spezifischer Marker für den LPR beschrieben [31]. Ebenso wird Pepsin eine wesentliche Rolle bei der Schädigung der laryngealen und pharyngealen Schleimhaut zugeschrieben, und es konnte auch gezeigt werden, dass hohe Pepsinspiegel im Speichel mit bestimmten endoskopischen Larynxbefunden und damit einem erhöhten RFS korrelieren [32]. Aktuell zeigen sich jedoch noch zahlreiche Schwierigkeiten beim Einsatz dieser Methodik. So konnten bisher deutliche tageszeitliche Schwankungen der Pepsinspiegel im Speichel festgestellt werden, wobei neuere Studien v. a. in den Morgenstunden die höchsten Konzentrationen von Pepsin im Speichel beschreiben [33]. Dennoch ist die Frage nach dem optimalen Zeitpunkt der Probenentnahme nicht abschließend geklärt. Ebenfalls zeigen sich die bislang zu dieser Thematik durchgeführten Studien sehr heterogen. Dies betrifft v. a. die Art und Weise der Probenentnahme, bei der Speichel, Abstriche oder auch pharyngeale Schleimhautbiopsien auf Pepsin analysiert wurden. Validierte und anerkannte Grenzwerte, ab denen von einem Refluxgeschehen ausgegangen werden kann, gibt es zum jetzigen Zeitpunkt noch keine. Ebenfalls erscheint gemäß neueren Studien eine Differenzierung zwischen dem LPR und GERD mit dieser Methodik nicht vielversprechend zu sein [28, 34].

Dennoch kann in Zusammenschau der verfügbaren Daten und auch im Hinblick auf die relativ einfache und günstige Durchführbarkeit davon ausgegangen werden, dass sich Pepsinmessungen im Speichel in der Zukunft zu einem ergänzenden Baustein in der Diagnostik des LPR entwickeln könnten [34].

## Fazit für die Praxis

- Aufgrund der heterogenen Studienlage und dem Fehlen eines Goldstandards bleibt die Diagnosestellung des LPR weiterhin schwierig und umstritten.
- Die Diagnosestellung des LPR kann jedoch als wahrscheinlich betrachtet werden, wenn/bei:
  - Anamnese suggestiv mit erhöhten Scores im RSI,
  - entsprechenden endoskopischen Befunden mit pathologischem RFS,
  - auffälliger ösophagealer oder oropharyngealer 24-h-pH-Metrie,
  - keine Hinweise auf eine andere Grunderkrankung für die Diagnose.
- Neue Fragebögen wie der RSS oder RSA könnten sich genauso wie die Messung der Pepsinkonzentration im Speichel zu vielversprechenden neuen Werkzeugen in der Diagnostik des LPR entwickeln.

## References

[1] Koufman JA (1991). The otolaryngologic manifestations of gastroesophageal reflux disease (GERD): a clinical investigation of 225 patients using ambulatory 24-hour pH monitoring and an experimental investigation of the role of acid and pepsin in the development of laryngeal injury. Laryngoscope.

[2] Lechien JR, Akst LM, Hamdan AL (2019). Evaluation and management of laryngopharyngeal reflux disease: state of the art review. Otolaryngol Head Neck Surg.

[3] Connor NP, Palazzi-Churas KL, Cohen SB (2007). Symptoms of extraesophageal reflux in a community-dwelling sample. J Voice.

[4] Kamani T, Penney S, Mitra I (2012). The prevalence of laryngopharyngeal reflux in the English population. Eur Arch Otorhinolaryngol.

[5] Borges LF, Chan WW, Carroll TL (2019). Dual pH probes without proximal esophageal and pharyngeal impedance may be deficient in diagnosing LPR. J Voice.

[6] Amarasiri DL, Pathmeswaran A, De Silva HJ (2013). Response of the airways and autonomic nervous system to acid perfusion of the esophagus in patients with asthma: a laboratory study. BMC Pulm Med.

[7] Chen Z, Sun L, Chen H (2018). Dorsal vagal complex modulates neurogenic airway inflammation in a guinea pig model with esophageal perfusion of HCl. Front Physiol.

[8] Runggaldier D, Pohl D (2017). Chronischer Husten – Welchen Platz haben PPIs?. Inf Arzt.

[9] Belafsky PC, Postma GN, Koufman JA (2002). Validity and reliability of the reflux symptom index (RSI). J Voice.

[10] Schindler A, Mozzanica F, Ginocchio D (2010). Reliability and clinical validity of the Italian Reflux Symptom Index. J Voice.

[11] Lechien JR, Huet K, Finck C (2017). Validity and reliability of a French version of reflux symptom index. J Voice.

[12] Lechien JR, Schindler A, Hamdan AL (2018). The development of new clinical instruments in laryngopharyngeal reflux disease: the international project of young otolaryngologists of the International Federation of Oto-rhino-laryngological Societies. Eur Ann Otorhinolaryngol Head Neck Dis.

[13] Lechien JR, Rodriguez Ruiz A, Dequanter D (2020). Validity and reliability of the reflux sign assessment. Ann Otol Rhinol Laryngol.

[14] Belafsky PC, Postma GN, Koufman JA (2001). The validity and reliability of the reflux finding score (RFS). Laryngoscope.

[15] Lechien JR, Bobin F, Muls V (2019). Gastroesophageal reflux in laryngopharyngeal reflux patients: clinical features and therapeutic response. Laryngoscope.

[16] Lee YC, Kwon OE, Park JM (2018). Do laryngoscopic findings reflect the characteristics of reflux in patients with laryngopharyngeal reflux?. Clin Otolaryngol.

[17] Johnson LF, Demeester TR (1974). Twenty-four-hour pH monitoring of the distal esophagus. A quantitative measure of gastroesophageal reflux. Am J Gastroenterol.

[18] Becker V, Graf S, Schlag C (2012). First agreement analysis and day-to-day comparison of pharyngeal pH monitoring with pH/impedance monitoring in patients with suspected laryngopharyngeal reflux. J Gastrointest Surg.

[19] Decalmer S, Stovold R, Houghton LA (2012). Chronic cough: relationship between microaspiration, gastroesophageal reflux, and cough frequency. Chest.

[20] Wenner J, Johnsson F, Johansson J (2007). Wireless esophageal pH monitoring is better tolerated than the catheter-based technique: results from a randomized cross-over trial. Am J Gastroenterol.

[21] Iluyomade A, Olowoyeye A, Fadahunsi O (2017). Interference with daily activities and major adverse events during esophageal pH monitoring with bravo wireless capsule versus conventional intranasal catheter: a systematic review of randomized controlled trials. Dis Esophagus.

[22] Andrews CN, Sadowski DC, Lazarescu A (2012). Unsedated peroral wireless pH capsule placement vs. standard pH testing: a randomized study and cost analysis. BMC Gastroenterol.

[23] Lawenko RM, Lee YY (2016). Evaluation of gastroesophageal reflux disease using the bravo capsule pH system. J Neurogastroenterol Motil.

[24] Ang D, Teo EK, Ang TL (2010). To Bravo or not? A comparison of wireless esophageal pH monitoring and conventional pH catheter to evaluate non-erosive gastroesophageal reflux disease in a multiracial Asian cohort. J Dig Dis.

[25] https://www.Restech.com/. Zugegriffen: 14.02.2021

[26] Yuksel ES, Slaughter JC, Mukhtar N (2013). An oropharyngeal pH monitoring device to evaluate patients with chronic laryngitis. Neurogastroenterol Motil.

[27] Wiener GJ, Tsukashima R, Kelly C (2009). Oropharyngeal pH monitoring for the detection of liquid and aerosolized supraesophageal gastric reflux. J Voice.

[28] Weitzendorfer M, Antoniou SA, Schredl P (2019). Pepsin and oropharyngeal pH monitoring to diagnose patients with laryngopharyngeal reflux. Laryngoscope.

[29] Mazzoleni G, Vailati C, Lisma DG (2014). Correlation between oropharyngeal pH-monitoring and esophageal pH-impedance monitoring in patients with suspected GERD-related extra-esophageal symptoms. Neurogastroenterol Motil.

[30] Desjardin M, Roman S, Des Varannes SB (2013). Pharyngeal pH alone is not reliable for the detection of pharyngeal reflux events: a study with oesophageal and pharyngeal pH-impedance monitoring. United Eur Gastroenterol J.

[31] Johnston N, Knight J, Dettmar PW (2004). Pepsin and carbonic anhydrase isoenzyme III as diagnostic markers for laryngopharyngeal reflux disease. Laryngoscope.

[32] Wang L, Liu X, Liu YL (2010). Correlation of pepsin-measured laryngopharyngeal reflux disease with symptoms and signs. Otolaryngol Head Neck Surg.

[33] Na SY, Kwon OE, Lee YC (2016). Optimal timing of saliva collection to detect pepsin in patients with laryngopharyngeal reflux. Laryngoscope.

[34] Calvo-Henriquez C, Ruano-Ravina A, Vaamonde P (2017). Is pepsin a reliable marker of laryngopharyngeal reflux? A systematic review. Otolaryngol Head Neck Surg.

